# Effect of Six-Week Resistance and Sensorimotor Training on Trunk Strength and Stability in Elite Adolescent Athletes: A Randomized Controlled Pilot Trial

**DOI:** 10.3389/fphys.2022.802315

**Published:** 2022-03-17

**Authors:** Steffen Mueller, Juliane Mueller, Josefine Stoll, Frank Mayer

**Affiliations:** ^1^Physiotherapy, Exercise Science and Applied Biomechanics, Department Computer Science – Therapy Sciences, Trier University of Applied Sciences, Trier, Germany; ^2^University Outpatient Clinic, Sports Medicine and Sports Orthopaedics, University of Potsdam, Potsdam, Germany

**Keywords:** core, training intervention, trunk stability, exercise, perturbation

## Abstract

Intervention in the form of core-specific stability exercises is evident to improve trunk stability. The purpose was to assess the effect of an additional 6 weeks sensorimotor or resistance training on maximum isokinetic trunk strength and response to sudden dynamic trunk loading (STL) in highly trained adolescent athletes. The study was conducted as a single-blind, 3-armed randomized controlled trial. Twenty-four adolescent athletes (14f/10 m, 16 ± 1 yrs.;178 ± 10 cm; 67 ± 11 kg; training sessions/week 15 ± 5; training h/week 22 ± 8) were randomized into resistance training (RT; *n* = 7), sensorimotor training (SMT; *n* = 10), and control group (CG; *n* = 7). Athletes were instructed to perform standardized, center-based training for 6 weeks, two times per week, with a duration of 1 h each session. SMT consisted of four different core-specific sensorimotor exercises using instable surfaces. RT consisted of four trunk strength exercises using strength training machines, as well as an isokinetic dynamometer. All participants in the CG received an unspecific heart frequency controlled, ergometer-based endurance training (50 min at max. heart frequency of 130HF). For each athlete, each training session was documented in an individual training diary (e.g., level of SMT exercise; 1RM for strength exercise, pain). At baseline (M1) and after 6 weeks of intervention (M2), participants’ maximum strength in trunk rotation (ROM:63°) and flexion/extension (ROM:55°) was tested on an isokinetic dynamometer (concentric/eccentric 30°/s). STL was assessed in eccentric (30°/s) mode with additional dynamometer-induced perturbation as a marker of core stability. Peak torque [Nm] was calculated as the main outcome. The primary outcome measurements (trunk rotation/extension peak torque: con, ecc, STL) were statistically analyzed by means of the two-factor repeated measures analysis of variance (*α* = 0.05). Out of 12 possible sessions, athletes participated between 8 and 9 sessions (SMT: 9 ± 3; RT: 8 ± 3; CG: 8 ± 4). Regarding main outcomes of trunk performance, experimental groups showed no significant pre–post difference for maximum trunk strength testing as well as for perturbation compensation (*p* > 0.05). It is concluded, that future interventions should exceed 6 weeks duration with at least 2 sessions per week to induce enhanced trunk strength or compensatory response to sudden, high-intensity trunk loading in already highly trained adolescent athletes, regardless of training regime.

## Introduction

A relevant task of the trunk is the compensation of external forces and loads to ensure the stability as well as the performance of the trunk or the entire body in everyday life and in high-performance sports (Cresswell et al., 1994; Hodges et al., 2001; Kibler et al., 2006; Borghuis et al., 2008; Hibbs et al., 2008; Calatayud et al., 2015). To protect the spine from repetitive and sudden excessive loads, an enhanced trunk stability is described as beneficial (Kibler et al., 2006; Borghuis et al., 2008; Hibbs et al., 2008; Wilson et al., 2014; Calatayud et al., 2015). Conversely, reduced trunk stability is considered a risk factor for the development of low back pain and lower extremity injuries, and also impairs athletic performance (Zazulak et al., 2007). The definition of trunk stability remains controversial. Trunk stability is defined by the ability to maintain “trunk balance” despite external mechanical forces or “neuro-muscular failure.” (Granata and England, 2006; Reeves et al., 2007, 2011; Reeves and Cholewicki, 2009). Although the concept of trunk stability is rather vague, there is strong evidence that strength capacity as well as sensorimotor control are relevant factors for a rapid compensation of external loading and perturbations, especially in high-performance sport (Gruber and Gollhofer, 2004; Gruber et al., 2006; Kibler et al., 2006; Gruther et al., 2009; Choi et al., 2010; Wirth et al., 2016). Besides, the trunk performance here represents the overall functional performance capacity including both, strength capacity as well as stability as response to sudden dynamic, high-intensity trunk loading induced by external perturbations. The term is used to summarize the different functional areas that contribute to the motoric performance capacity of the trunk.

Intervention in the form of active exercises is evident to improve trunk stability (Wang et al., 2012; Saragiotto et al., 2016; Wirth et al., 2016; Mueller and Niederer, 2020; Niederer et al., 2020; Niederer and Mueller, 2020). Various strengthening exercises have been used and have been shown to be effective (Hibbs et al., 2008; Stuber et al., 2014). Significantly, in addition to the focus on trunk strengthening exercises, further training methods involving neuromuscular, sensorimotor training or combinations of these have emerged in the last years (Saragiotto et al., 2016; Arampatzis et al., 2017; Mueller et al., 2018b; Niederer et al., 2020). Core-specific sensorimotor exercises are an effective method to improve the neuromuscular activity of the trunk musculature and consequently improve trunk stability (Arampatzis et al., 2017; Mueller et al., 2018a,b). Sensorimotor training emphasizes activation of the deep trunk muscles (Hodges and Moseley, 2003), improves muscle control, and enhances inter- and intramuscular coordination (Gruber and Gollhofer, 2004). In particular, for resistance training and sensorimotor training, the benefits in terms of maximal eccentric and concentric trunk strength and peak torque in sudden dynamic trunk loading (STL) situations have not been systematically elucidated, while the differential effect of resistance and sensorimotor training remains an open question. Optimizing trunk stability and trunk strength contains the greatest potential for preventive effects. Even though adaptations to strength training are generally evident after eight to twelve weeks of training, Mueller et al. (2018b) proofed significant enhancements in high-intensity trunk loading response (to sudden perturbations) in healthy, well-trained adults already after 6 weeks of resistance or sensorimotor training. It remains unclear if this training regime (two sessions per week á 1 h for 6 weeks) could also lead to similar improvements in adolescent athletes, as there is existing evidence that even in adolescents 6 weeks of resistance training are evident to enhance upper and lower body performance (Faigenbaum et al., 2007).

Consequently, the aim of this study was to investigate and compare the effect of a six-week sensorimotor and a resistance training program on maximum isokinetic trunk strength and response (peak torque) to sudden high-intensity trunk loading. Improvement in isokinetic trunk strength and response to sudden high-intensity trunk loading was expected for participants in the two experimental groups compared to the control group.

## Materials and Methods

### Study Design

The study was conducted as a single-blind (investigator), 3-armed randomized controlled trial with 6-week intervention phase and two measurement days pre/post-intervention (M1/M2). Participants were allocated to either the two experimental groups, which received sensorimotor training (SMT) or resistance training (RT), or the control group (endurance training).

**Figure 1:**
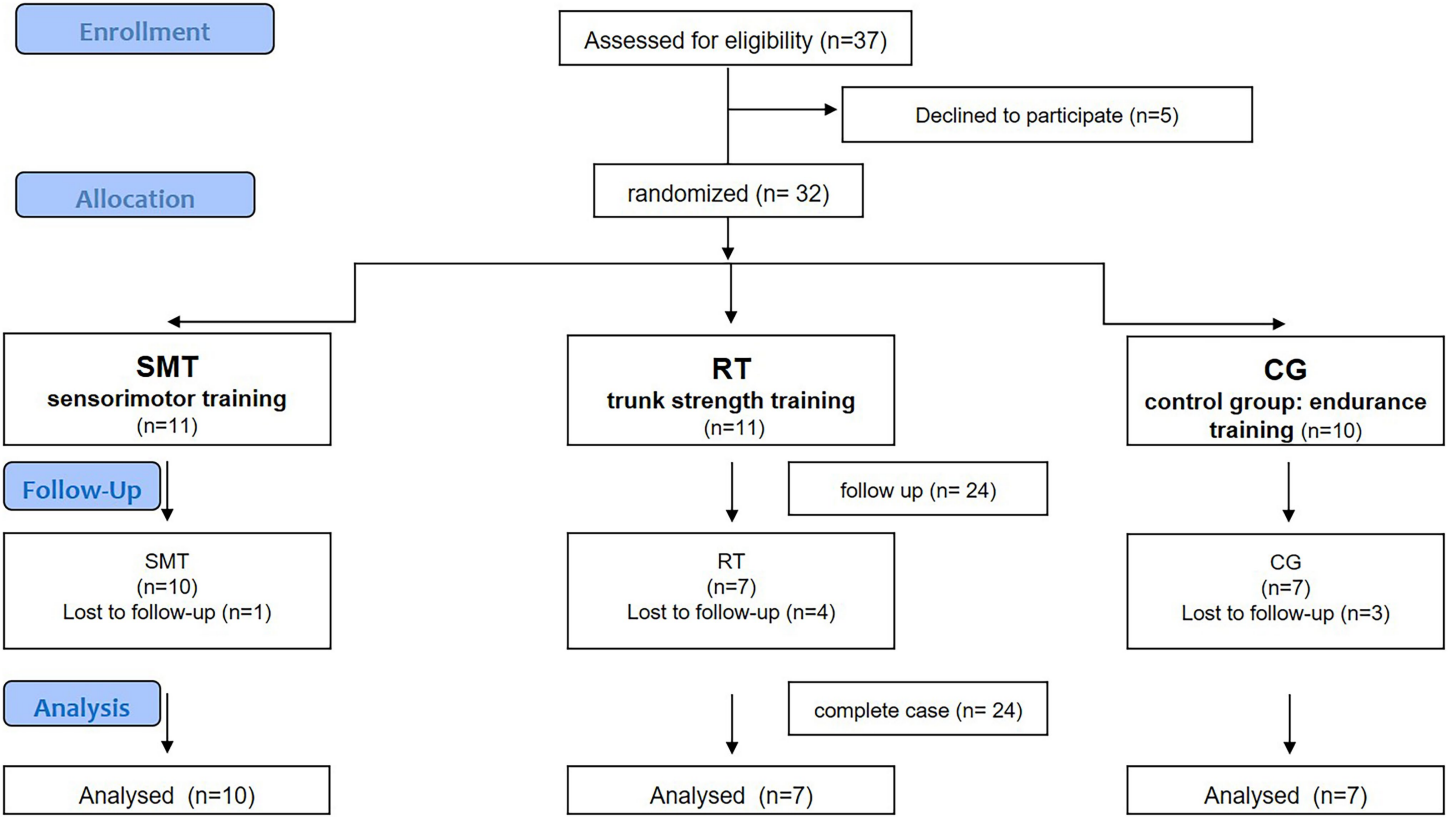
Study flowchart.

The study was registered at the German Clinical Trial Register (DRKS Trial Registration No.: DRKS00000776). Potential participants were screened and examined by a sports medicine physician to determine eligibility before baseline assessment and randomization to the intervention groups (Figure 1).

### Participants

Healthy adolescent elite athletes from the elite schools of sports of the federal state of Brandenburg (Germany) were recruited *via* the university outpatient clinic (e.g., athletes receiving annual health check-ups) and existing contacts with training groups at the Olympic Center. Elite schools of sports are special types of school, which ensure talented young elite athletes will be encouraged to their full potential and will also attain their educational qualifications. Inclusion criteria were an age between 11 and 17 years, both genders and being an elite athlete at the elite school of sport. Exclusion criteria were acute infection, pregnancy, any illness that would contraindicate exercise, and (low) back pain. All participants and their legal guardian were informed of the study and the specific testing procedures in a personal conversation with the principal investigator and through written study information during their stay at the university outpatient clinic. Before voluntary participation in the study, the legal guardians and the children provided written informed consent. The University Ethical committee approved all procedures conducted during the study.

Thirty-two adolescent athletes were included in the study and randomly assigned into SMT, RT, and CG, with *n* = 24 participants eligible for final analysis (Figure 1). The randomization list, generated by “randomization.com,” was kept in a locked cabinet. A research assistant not involved in the outcome assessment revealed the group allocation. Participants’ baseline characteristics are displayed in Table 1, separated by intervention and control groups. There was no statistically significant difference in any of the baseline characteristics between the groups (*p* > 0.05).

**Table 1:** Anthropometric and training characteristics of the study participants at baseline for control (CG), resistance training (RT) and sensorimotor training (SMT) groups [mean ± SD].

Group	n (m/f)	Age [yrs]	Body mass [kg]	Body height [cm]	Sport disciplines [n]	Training-volume [h/week]	Back pain begin of measurement day [VAS; cm]
CG (*n*=7)	3/4	16 ± 1	68 ± 10	181 ± 11	Triathlon: *n*=2 Rowing: *n*=3 Canoeing: *n*=2	22 ± 11	0.6 ± 0.9
SMT (*n*=10)	5/5	16 ± 1	65 ± 10	179 ± 11	Triathlon: *n*=3 Rowing: *n*=4 Canoeing: n=3	24 ± 6	0.3 ± 0.8
RT (*n*=7)	2/5	16 ± 1	71 ± 13	175 ± 9	Triathlon: *n*=3 Rowing: *n*=2 Canoeing: *n*=2	22 ± 8	0.4 ± 0.5

CG, control group; SMT, sensorimotor training group; RT, resistance training group.

### Intervention

Participants in both intervention groups (SMT; RT) were instructed to perform standardized, center-based training for 6 weeks, 2 times per week, at a duration of 60 min each session. Intervention groups consisted of 3 to 4 participants and were instructed by experienced therapists. Both interventions had matched overall training volumes and started with a 5-min general physical warm-up using different exercises, such as jumping jacks.

**Figure 2:**
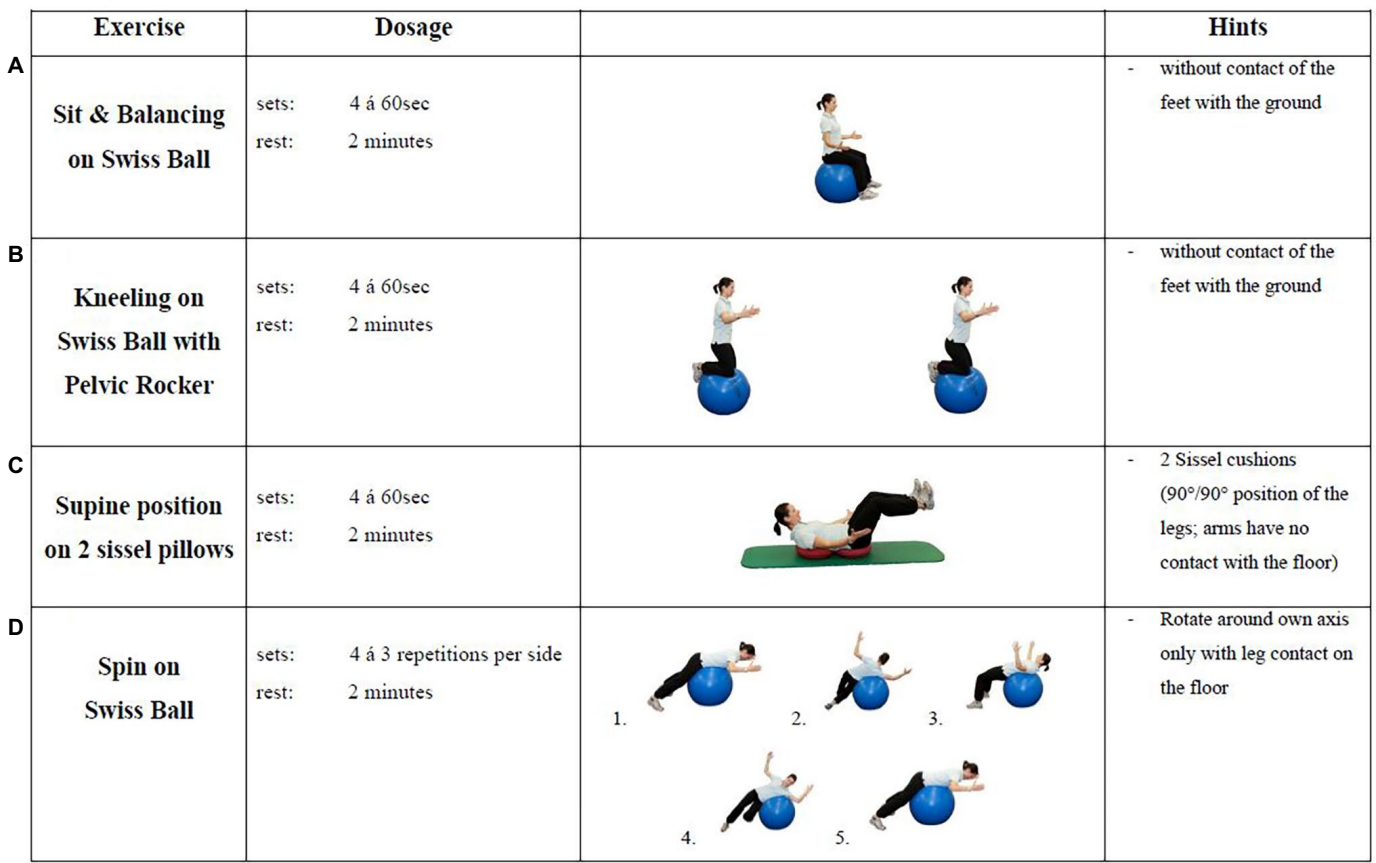
Sensorimotor training (SMT) intervention exercises.

SMT consisted of four different core-specific sensorimotor exercises using instable surfaces (Figure 2). Each type of stabilizing exercise was carried out for 60 s with 4 sets. Rest between sets and between the tasks was standardized to 2 min. The exercise level was adapted on an individual basis every week. This was done by the therapist by increasing the difficulty of the four basic exercises, for example, by adding unstable surfaces or additional movement task. All athletes in the SMT group received verbal feedback from the therapists focusing on movement quality and error correction while performing the exercises.

We applied maximal strength training, in line with recommendations for general progressive strengthening (American College of Sports Medicine, 2009). Due to the 6-week intervention, improvements would be linked predominantly to adaptations of the intramuscular coordination. RT consisted of four trunk strength exercises using strength training machines for lateral flexion and rotation (Extension Bench and Torso Rotation, Cybex International, Inc. United States), as well as an isokinetic dynamometer for flexion and extension (CON-TREX MJ/TP 1000, Physiomed Elektromedizin AG, Germany; Mueller et al., 2018b). All strengthening exercises were executed at moderate velocity for three sets of eight repetitions and an intensity of 85% of the individual’s maximum strength capacity. The rest period between the sets and exercises was 2 min. The intensity (85%) of the rotation and lateral flexion strengthening exercises was determined by means of the 1-repetition maximum (1RM) method. Trunk flexion and extension were trained in eccentric mode (30°/s; ROM: 55°), and intensity (85%) was determined using the isokinetic maximum strength test. The intensity was redefined every 2 weeks to ensure an individualized, progressive resistance training (1RM; maximum isokinetic strength test). All athletes received verbal feedback on the quality of movement execution for exercise 1 and 2, as well as any necessary error correction. For the execution of exercise 3 and 4 in the isokinetic dynamometer, visual biofeedback on the achievement of 85% intensity from the individual maximum strength test was provided.

All participants in the CG received an unspecific heart frequency controlled endurance training (50 min at max. Heart frequency of 130 HF) on a bicycle ergometer, treadmill ergometer, or arc trainer (randomized ergometer).

For each athlete, each training session was documented in an individual training diary with the most important information about each training session (e.g., level of SMT exercise; 1RM for strength exercise, pain). Low back pain was monitored by use of a VAS (0–10 cm) in regular intervals throughout each training session. Besides, all participating athletes followed their regular training routines Table 1 with the presented hour of training per weeks next to the applied intervention.

### Experimental Protocol and Outcome Measures

Moreover, the procedures of the experimental protocol were described elsewhere (Mueller et al., 2018b). However, the experimental protocol at both assessment point (M1/M2) was identically: Initially, anthropometrics and training habits [overall training time (h/week), sports discipline] were assessed. Afterward, all participants were screened and examined by a sports medicine physician to determine eligibility before baseline assessment followed by randomization to the intervention groups and/or experimental protocol. All participants were assessed before intervention (baseline = M1) and after intervention (post = M2) by a blinded assessor. At both assessment points (M1/M2), outcomes were measured in the following order: back pain, isokinetic trunk rotation strength, response to sudden, high-intensity trunk rotation loading, isokinetic trunk extension strength, response to sudden, high-intensity trunk extension loading and, finally, back pain was re-assessed.

#### Back Pain

Back pain intensity was measured, as a control parameter to account for pain or injury development, at rest and after high-intensity strength testing protocol on a 10 cm visual analogue scale [VAS (cm)].

#### Isokinetic Trunk Strength

Trunk rotator and extensor isokinetic concentric and eccentric strength were assessed with an isokinetic dynamometer. All participants underwent a general physical warm-up of at least 10 min on a treadmill before isokinetic testing. For (right-sided) trunk rotation strength testing, participants were placed before an angular dynamometer (CON-TREX WS, Physiomed Elektromedizin AG, Germany) in a seated position, with a rotational range of motion of 63° (Mueller et al., 2018b). Trunk strength measurements for extension were performed in a standing position (CON-TREX MJ/TP 1000, Physiomed Elektromedizin AG, Germany). Participants were fixed to the dynamometer with adjustable adapters at the lower leg and knee, as well as two non-stretching belts at the hip and upper body. The range of motion was set to 55° (Mueller et al., 2018b). Trunk strength measurements included an additional 60s warm-up and familiarization trial for each test situation, performed at a moderate intensity. Additionally, preceding all measurements, an identical practice trial with submaximal effort was performed. Resting time between the warm-up and each maximum strength test was standardized to a minimum of 1 min. Maximum strength in rotation and extension was tested in concentric (30°/s, con) and eccentric (30°/s, ecc) modes, performing five repetitions.

#### Response to Sudden, High-Intensity Trunk Loading

Sudden dynamic trunk loading was applied as a represent for trunk stability. Sudden, high-intensity trunk loading was induced during an additional eccentric mode (30°/s) by means of a superimposed customized perturbation (acceleration from 30°/s to 330°/s within 120 ms for trunk rotation and 150°/s within 250 ms for trunk extension; STL).

Verbal encouragement was given throughout the entire test to ensure participants’ maximum effort. The outcome measurements analyzed for all test modes were peak torque [Nm] in trunk extension (Ext) and trunk rotation (Rot), calculated as the mean of the three peak torque values from five repetitions (Müller et al., 2007). The reproducibility of the novel STL test was proven in a prior pilot study (STL: ICC: 0.94, test–retest variability: 8.53 ± 6.33%; bias±1.96SD: 8.16 ± 64.8 Nm; *n* = 10; Engel et al., 2013; Mueller et al., 2018b).

### Data Analysis

All data were documented in a case report form if not captured by a computer. The data were transferred, manually from the case report forms (CRF), into a database for further statistical analysis. For all data, a plausibility check was performed. Further analyses followed the intention-to-treat principle. Statistical analysis was done descriptively (means ± standard deviation (SD), means with upper/lower 95% confidence interval (CI)) for baseline, post-intervention test (M1, M2), and the pre–post difference. The primary outcome measurements (trunk rotation/extension peak torque: con, ecc, STL) were statistically analyzed by means of the 2-factor repeated measures analysis of variance ANOVA (*α* = 0.05; JMP, SAS Institute^®^).

A power analysis to calculate sample size was not performed. The number of subjects to be included was based on previous published studies with comparable outcomes (Arampatzis et al., 2017; Mueller et al., 2018b). *N* = 20 subjects per group will be included. Mueller et al. (2018b) estimated a minimum sample size of 14 participants per group.

## Results

### Flow and Characteristics of Participants Through the Study

Of the 37 participants that were screened, all met the inclusion criteria, five declined participation and 32 participants were therefore included in the study. One participant in the SMT group, four athletes out of the RT group and three athletes out of the CG did not complete the study (Figure 1). The loss of participants was especially due to the missing of measurement M2. Rescheduled time windows for final measurement (M2) were also not met without giving reasons. No specific pattern between the three groups could be identified for the non-appearance or cancellation of the second measurement. Therefore, 24 adolescent athletes (14f/10 m; 16 ± 1 yrs.; 178 ± 10 cm; 67 ± 11 kg; training sessions/week 15 ± 5; training h/week 22 ± 8) were included into final analysis. The baseline characteristics (anthropometrics, training data, outcomes) of the three groups are shown in Table 1. There are no statistical significant differences (*p* > 0.05) between the three groups for all baseline characteristics (age, body height/weight, training hours per week). Regular training routine of all athletes included athletic (resistance and endurance) as well as technical (sport-specific) training parts. The precentral distribution of these parts did not differ significantly between the two experimental and the control group (*p* > 0.05). The athletes included into the SMT as well as in the RT group reported a distribution of 58% endurance, 30% resistance, and 12% technical training. The athletes in the CG group reported a distribution of 65% endurance, 25% resistance and 10% technical training for their regular training.

### Training Compliance

Training documentation revealed on average 8 (SD: ±3) executed training sessions by the RT group, 9 (SD: ±3) by the SMT group and 8 (SD: ±4) by the CT group out of a maximum of 12 possible training sessions. Overall, this resulted in 1.4 ± 0.5 sessions per week for all participating athletes (training session/week: SMT: 1.5 ± 0.5; RT: 1.3 ± 0.5; C: 1.4 ± 0.6).

### Back Pain

Back pain intensity did not change between the two measurement days M1 and M2 for any of the three groups (e.g., VAS (at the beginning of each measurement day): SMT 0.3 ± 0.8 (M1)/0.3 ± 0.6 (M2); RT: 0.4 ± 0.5 (M1)/0.7 ± 1.1 (M2); CG: 0.6 ± 0.9 (M1)/06. ± 1.5 (M2); *p* > 0.05).

### Main Outcomes (Isokinetic Strength/STL)

**Figure 3:**
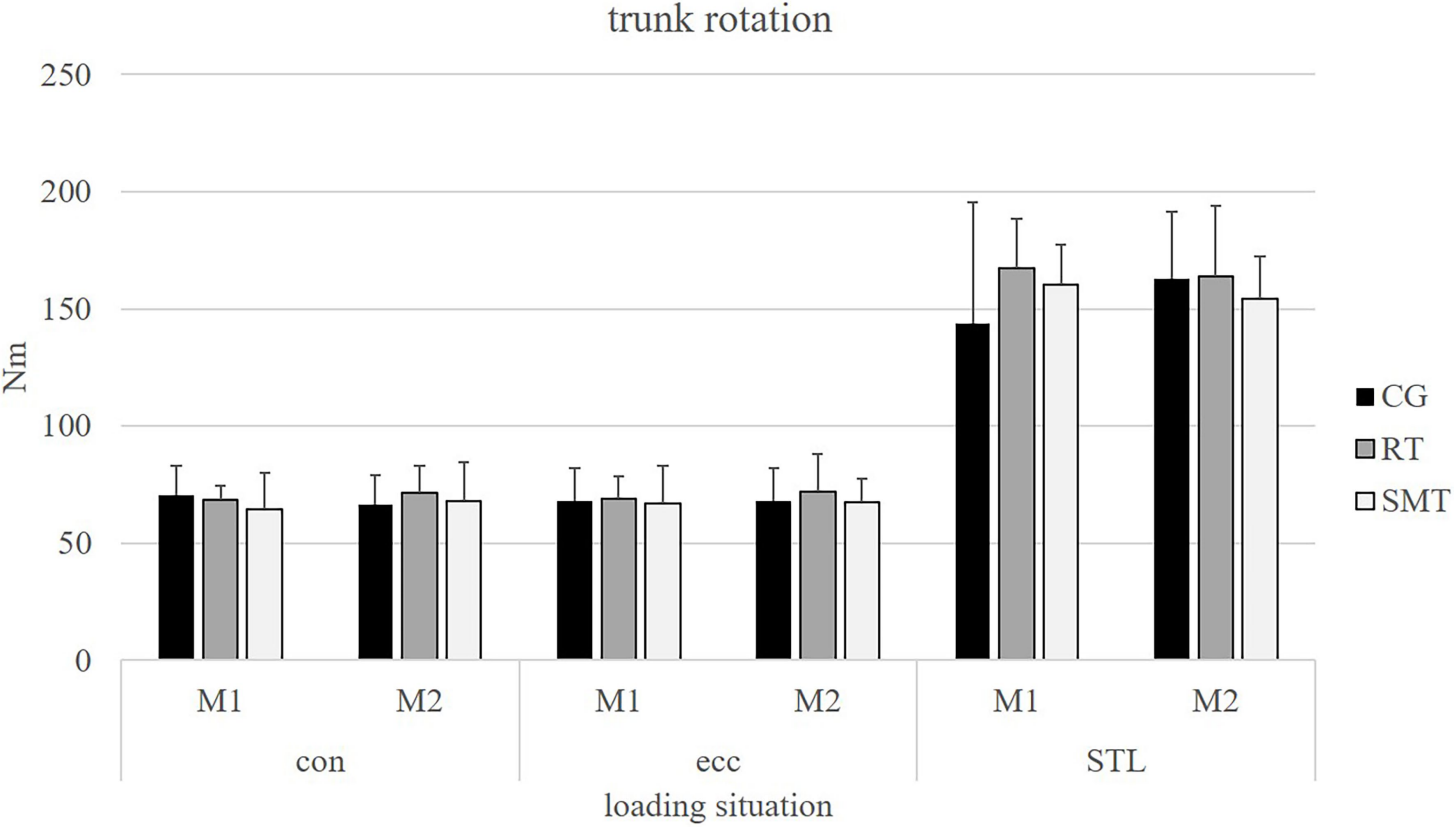
Absolute values of mean (mean ± SD) peak torque [Nm] for baseline (M1) and post-intervention measurements (M2) for each group in trunk rotation isokinetic concentric, eccentric, and sudden trunk loading (STL) testing.

**Figure 4:**
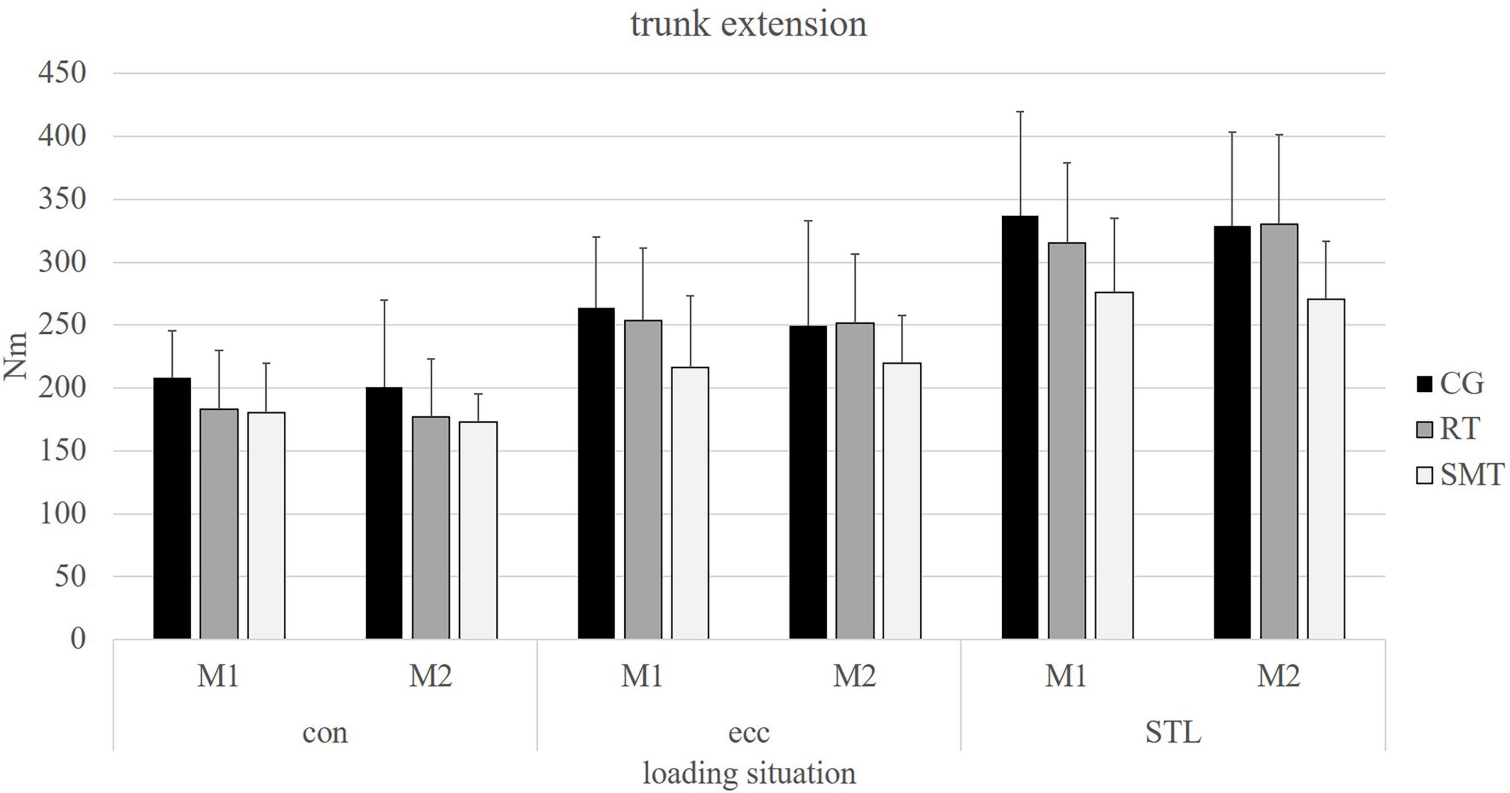
Absolute values of mean (mean ± SD) peak torque [Nm] for baseline (M1) and post-intervention measurements (M2) for each group in trunk extension isokinetic concentric, eccentric, and sudden trunk loading (STL).

Results of the isokinetic strength and STL testing for trunk rotation as well as trunk extension (M1/M2) are presented in Table 2 and displayed in Figures 3, 4 for all three groups. No significant differences were present over time (M1/M2; *p* > 0.05) for all outcome measures in any of the groups.

**Table 2:** Absolute values of mean (95% CI) peak torque [Nm] for baseline (M1) and post-intervention measurements (M2) for each group in trunk rotation and extension for isokinetic concentric, eccentric and sudden trunk loading (STL).

Outcome	Day	Groups					
		CG		RT		SMT	
		Mean	(95% CI)	Mean	(95% CI)	Mean	(95% CI)
**Trunk rotation**							
con	M1	70	(59–82)	69	(62–75)	64	(53–76)
	M2	66	(55–78)	71	(59–84)	68	(57–80)
ecc	M1	68	(55–81)	69	(59–79)	67	(55–78)
	M2	68	(56–81)	72	(55–89)	67	(60–75)
STL	M1	144	(90–198)	168	(141–194)	160	(148–173)
	M2	163	(137–189)	164	(126–201)	155	(141–169)
**Trunk extension**							
con	M1	208	(173–243)	183	(140–226)	181	(153–209)
	M2	201	(137–265)	177	(135–219)	173	(157–189)
ecc	M1	264	(212–316)	253	(200–307)	217	(177–257)
	M2	250	(173–327)	251	(200–302)	220	(192–247)
STL	M1	337	(261–414)	315	(256–374)	276	(234–318)
	M2	329	(260–398)	330	(264–396)	270	(237–304)

Regarding main outcomes of trunk performance (isokinetic strength/STL), no statistically significant differences could be observed between the intervention and control groups (*p* > 0.05). No significant group by time interaction (*p* > 0.05) could be observed for all presented outcome measures. The power (*post hoc* power analysis) of the outcomes reached between 0.06 (STL in rotation) to 0.34 (STL in extension) for the RT group and between 0.05 (eccentric testing in rotation) to 0.33 (concentric testing in rotation) for the SMT group.

## Discussion

The primary aim of this randomized controlled trial was to enhance trunk strength capacity as well as response to sudden high-intensity loading, known to be a relevant risk factors for back pain (Hodges and Moseley, 2003; Borghuis et al., 2008), through a 6-week sensorimotor or resistance training in elite adolescent athletes. The results suggest that two additional sessions of sensorimotor or resistance training per week over 6 weeks are not sufficient to improve trunk strength or compensatory response to sudden, high-intensity trunk loading in already highly trained adolescent athletes.

With regards to the primary outcomes of trunk performance, both experimental groups (SMT/RT) showed no significant pre–post difference for maximum strength in concentric and eccentric testing for trunk rotation as well as for trunk extension. In addition, the results presented show that neither training program increased peak torque in response to sudden, high-intensity trunk loading. However, this is in contrast to previous results reported for adult (recreational and elite) athletes (Arampatzis et al., 2017; Mueller et al., 2018b). It has to be discussed whether the applied STL test on the isokinetic dynamometer is a suitable measurement situation for the assessment of dynamic trunk stability. As the applied sensorimotor intervention consists of four exercises that address rather directly the trunk stability but not directly the balance ability in case of static upright postural control of the entire body, an isolated trunk stability test can be considered reasonable (Mueller et al., 2018b). Moreover, Mueller et al. (2018b) could proof a statistically significant improvement for peak torque in response to sudden high-intensity trunk loading with a comparable study design and test setup. The reason for these shown differences could on the one hand be that the already high training volume of the adolescent athletes, leading to rather unexpectedly small strength gains in isokinetic mode. This is supported by the condition of reduced remaining adaptation capacity with increased training level also known from elite adult athletes. On the other hand, Mueller et al. (2018b) prescribed three training sessions per week. Therefore, it has to be discussed, that the prescribed dosage of two sessions per week in our study was too low for these already highly trained adolescent athletes with a weekly training level of more than 20 h. In this context, the discussion of athletes’ adherence to the intervention can possibly be used as an additional explanation. The adolescent athletes in this study showed a limited adherence (70%) to the implementation of an additional intervention for two sessions per week. Although two sessions per week were prescribed, the athletes only attended an average of 1.3 to 1.5 sessions per week. In this context, it additionally has to be discussed if an intervention duration of 6 weeks (with two sessions per week) may have been too short to be able to achieve neuromuscular adaptations and strength gains (Lesinski et al., 2016). Arampatzis et al. (2021) implemented a perturbation-based exercise intervention with a large proportion of sensorimotor exercises for two times per week á 25 min over a period of 1 year in adolescent athletes aged 13–18 yrs. The authors were able to proof significant increase of trunk extensor and flexor strength (Arampatzis et al., 2021). Moreover, Lesinski et al. (2016) reported in their systematic review that short-term resistance training is already effective in adolescent athletes and should last 9–12 weeks.

In addition, when comparing the intervention programs, similar results in trunk isokinetic strength and STL were not expected for the two experimental groups. In this context, it is relevant to elucidate whether training adaptations approached differently (SMT and RT) lead to a similar response in the outcomes studied. (Gruber and Gollhofer, 2004; American College of Sports Medicine, 2009). Intervention adaptations after RT could be speculated to focus primarily on a muscular level, in contrast to a more neuronal component for SMT training, leading to the same functional outcome in the short term of 6 weeks (Gruber and Gollhofer, 2004; Taube et al., 2007; Ratamess et al., 2016). For both interventions, but especially for RT, we expected higher effects on the isokinetic strength outcomes, since the exercise programs were designed with valid training volumes, intensities, and duration (American College of Sports Medicine, 2009). It could be speculated that the already well-trained participants would have needed an even higher training amount to additionally adapt to on top of their high baseline trunk strength.

Core-specific exercise programs for the treatment and prevention of chronic non-specific low back pain can improve trunk performance, leading to a reduction in the recurrence of low back pain or pain relief (Hibbs et al., 2008; Choi et al., 2010; Mannion et al., 2012). However, studies are limited and assess trunk stability most often indirectly *via* performance tests, isolated maximum strength tests, and muscular activity measurements (Leetun et al., 2004; Kibler et al., 2006; Borghuis et al., 2008; Hibbs et al., 2008). This may not apply to dynamic trunk stability in high-intensity loading situations that occur in high-performance sport. The test setup presented here accounts for this issue using STL. A higher peak torque response could be interpreted as a noticeable increase in reactive load compensation after intervention. In consequence, this should be interpreted as a complex neuromuscular response counteracting external sudden trunk loading. In light of the knowledge of differences in perturbation compensation responses for healthy persons compared to those with low back pain, the results might be meaningful not only for prevention, but also for rehabilitation strategies (Cholewicki et al., 2000; Radebold et al., 2000). In addition, the STL test appears to be feasible for the adolescent athletes studied, as no complaints were documented and no back pain occurred.

Certain limitations have to be taken into account when interpreting the results. The inclusion of male and female adolescent athletes may have increased variance and influenced the impact of the intervention. Besides, we did not assess the maturity status of each participant since we assume independently of this positive adaptations. However, the different individual stage of development might have influenced on the extend of adaptation. As mentioned above, the six-week resistance training seem to be too short to explore muscular adaptation mechanism. Furthermore, because the study focuses on measuring peak torque, it is not able to provide detailed physiological explanations at the neuronal level without adding, for example, surface electromyographic measurements (Gruber et al., 2006; Taube et al., 2007). The control group was not a passive control group due to the fact, that whole training groups were participating in the study. For ethical reasons, all athletes out of one training group, including those randomly assigned to the control group, had to be offered a physical activity. The choice of exercise content (endurance) for the control group was based on the knowledge that endurance training at a low heart rate (120 bpm) has no significant effects on maximum strength and neuromuscular control of the trunk. The low sample size has to be stated as a major limiting factor, leading to a low power, of the study. Our results must thus be interpreted with care as explorative pilot findings and should be proven or disproven by future studies. Besides, the higher loss of participants in the RT and CG group as opposed to the SMT group may have influenced the results.

It can be concluded, that two additional bouts of sensorimotor or resistance training per week are not sufficient to improve trunk performance. Therefore, future interventions should exceed 6 weeks duration with at least two sessions per week to induce enhanced trunk strength or compensatory response to sudden, high-intensity trunk loading in already highly trained adolescent athletes, regardless of training regime. The high-intensity sudden trunk loading (STL) protocol seems to be certainly feasible and valid for the assessment of trunk stability in the adolescent elite athletes studied, as no back pain occurred. In addition, the validation of the presented intervention programs as well as the innovative trunk loading tests (STL) should also be performed in athletes with back pain in future research.

## Data Availability Statement

The original contributions presented in the study are included in the article, further inquiries can be directed to the corresponding authors.

## Ethics Statement

The studies involving human participants were reviewed and approved by University of Potsdam Ethic Commission. Written informed consent to participate in this study was provided by the participants’ legal guardian/next of kin. Written informed consent was obtained from the individual(s) for the publication of any potentially identifiable images or data included in this article.

## Author Contributions

The authors confirm contribution to the paper as follows: study conception and design: FM, SM, JM, JS. Data collection: SM, JS, JM. Analysis and interpretation of results: FM, SM, JM. Draft manuscript preparation: SM, JM. All authors contributed to the article and approved the submitted version.

## Funding

The present study was initiated and funded by the German Federal Institute of Sport Science. (Granted number: BISp IIA 1-080126/09-13).

## Conflict of Interest

The authors declare that the research was conducted in the absence of any commercial or financial relationships that could be construed as a potential conflict of interest.

## Publisher’s Note

All claims expressed in this article are solely those of the authors and do not necessarily represent those of their affiliated organizations, or those of the publisher, the editors and the reviewers. Any product that may be evaluated in this article, or claim that may be made by its manufacturer, is not guaranteed or endorsed by the publisher.
